# Transient ischemic stroke triggers sustained damage of the choroid plexus blood-CSF barrier

**DOI:** 10.3389/fncel.2023.1279385

**Published:** 2023-12-01

**Authors:** Yang Chen, Lin Lin, Mohammad Iqbal H. Bhuiyan, Kai He, Roshani Jha, Shanshan Song, Victoria M. Fiesler, Gulnaz Begum, Yan Yin, Dandan Sun

**Affiliations:** ^1^Department of Neurology, The Second Affiliated Hospital of Dalian Medical University, Dalian, Liaoning, China; ^2^Department of Neurology, University of Pittsburgh, Pittsburgh, PA, United States; ^3^The School of Pharmacy, University of Texas at El Paso, El Paso, TX, United States; ^4^Pittsburgh Institute for Neurodegenerative Diseases, University of Pittsburgh, Pittsburgh, PA, United States; ^5^Research Service, Veterans Affairs Pittsburgh Health Care System, Pittsburgh, PA, United States

**Keywords:** blood-CSF barrier, *Lcn2*, Na^+^ -K^+^ -Cl^–^ cotransporter, neuroinflammation, SPAK

## Abstract

Neuroinflammation is a pathological event associated with many neurological disorders, including dementia and stroke. The choroid plexus (ChP) is a key structure in the ventricles of the brain that secretes cerebrospinal fluid (CSF), forms a blood-CSF barrier, and responds to disease conditions by recruiting immune cells and maintaining an immune microenvironment in the brain. Despite these critical roles, the exact structural and functional changes to the ChP over post-stroke time remain to be elucidated. We induced ischemic stroke in C57BL/6J mice via transient middle cerebral artery occlusion which led to reduction of cerebral blood flow and infarct stroke. At 1–7 days post-stroke, we detected time-dependent increase in the ChP blood-CSF barrier permeability to albumin, tight-junction damage, and dynamic changes of SPAK-NKCC1 protein complex, a key ion transport regulatory system for CSF production and clearance. A transient loss of SPAK protein complex but increased phosphorylation of the SPAK-NKCC1 complex was observed in both lateral ventricle ChPs. Most interestingly, stroke also triggered elevation of proinflammatory *Lcn2* mRNA and its protein as well as infiltration of anti-inflammatory myeloid cells in ChP at day 5 post-stroke. These findings demonstrate that ischemic strokes cause significant damage to the ChP blood-CSF barrier, contributing to neuroinflammation in the subacute stage.

## Introduction

Choroid plexuses (ChP) are leaf-like structures located in all four ventricles of the brain, consisting of highly vascularized stroma surrounded by choroid plexus epithelial cells (CPECs) (Dziegielewska et al., 2001). In physiological conditions, tight junctions (TJs) and adherens junctions between CPECs maintain the blood-CSF barrier (BCSFB) integrity and prevent the free passage of large hydrophilic molecules and cells paracellularly between the CSF and blood interface (Redzic and Segal, 2004; Solár et al., 2020). Thus, the ChP plays an important role in the neuroinflammation process and is involved in many diseases including systemic inflammation, stroke, traumatic brain injury (TBI), neurodegenerative diseases, and autoimmune disorders of the CNS (Solár et al., 2020). The ChP is also known to be one of the lymphocyte infiltration routes in post-stroke brains (Llovera et al., 2017). Monocyte-derived macrophage levels were found to have increased in the ChP and CSF during the first week after ischemic insult (Ge et al., 2017). CPEC swelling (1–4 days), ChP edema (24 h), cell proliferation, cell death (6 h), and the disruption of the BCSFB (6 h) have been detected in stroke models of rats (Nagahiro et al., 1994; Gillardon et al., 1996; Li et al., 2002; Ennis and Keep, 2006). However, the underlying mechanisms of ChP structural and functional damage in stroke brains are not yet completely defined. The goal of this study is to investigate time-dependent changes of ChP structures and functions, particularly by examining changes of CPEC ion transport protein complexes and the BCSFB.

One well-known function of the ChP is to secrete CSF (Redzic and Segal, 2004). Human ChP produces approximately 500 mL of CSF per day (Hutton et al., 2021). A vital ion transporter for this process is the sodium-potassium-chlorine cotransporter isoform 1 (NKCC1), which is expressed abundantly on the apical membrane of CPECs (Gregoriades et al., 2019). Coupling with the function of the water channel AQP1, NKCC1-mediated Na^+^, K^+^, Cl^–^ flux has been posited to contribute to the CSF secretion (Steffensen et al., 2018; Blazer-Yost, 2023). Pathological stimulation of ChP SPAK-NKCC1 protein complex causes LPS- or hemorrhagic hydrocephalus in adult rats (Robert et al., 2023). Toll-like receptor 4 (TLR4) and nuclear factor kappa B (NF-κB)-dependent activation of SPAK-NKCC1 complex has also been shown to mediate CSF hypersecretion in post-infectious hydrocephalus or pediatric post-hemorrhagic hydrocephalus (PHH) models (Karimy et al., 2017; Robert et al., 2023). However, in the PHH model, overexpression of the NKCC1 protein prevents ventriculomegaly by enhancing CSF clearance and CSF K^+^ uptake (Xu et al., 2021; Sadegh et al., 2023). This suggests that the direction of NKCC1-mediated electrolyte and water transport can be in the “reverse” mode which redistributes CSF ions via the CPEC uptake (Blazer-Yost, 2023). We previously reported that ischemic stroke in mice stimulates the CPEC apical membrane expression of SPAK-NKCC1 complex at 24 h post-ischemic stroke, which is associated with loss of the blood–CSF barrier integrity and increased immune cell infiltration into the ChP (Wang et al., 2022). However, whether these changes are sustained in the subacute phase at 1–7 days post-ischemic stroke remains unknown.

In this study, we report that ischemic stroke in C57BL/6J mice induced by transient middle cerebral artery occlusion triggers time-dependent changes in the blood-CSF barrier permeability, tight-junction damage and dynamic changes of SPAK-NKCC1 complex pathway at 1–7 days post-stroke, which is featured by an elevation of albumin permeability, phosphorylatory stimulation of SPAK-NKCC1 protein complex, elevation of proinflammatory lipocalin-2 (*Lcn2*) mRNA and protein expression, and elevation of CD11b^+^/CD45^+^/CD206^+^ macrophages in the ChP. These findings indicate that ischemic stroke caused sustained damage of the ChP blood-CSF barrier in the subacute stroke brains.

## Materials and methods

### Chemicals

Rabbit anti-SPAK/OSR1 (Cat# 2281S) and rabbit anti-NKCC1 antibody (Cat# 85403T) were purchased from Cell Signaling. Rabbit anti-pSPAK/pOSR1 (Cat# 07-2273) and rabbit anti-pNKCC1 (Cat# ABS1004) were from EMD Millipore. Rabbit anti-albumin was purchased from abcam (Cat# Ab19196). Mouse monoclonal NGAL antibody (LCN2) was from Santa Cruz Biotechnology (Cat# sc-515876). Collagenase type I and collagenase type IV were purchased from Worthington Biochemical Corporation (Cat# LS004196) and Thermo Scientific (Cat# 17104019), respectively. DNase I was purchased from Sigma Aldrich (Cat# 10104159001). Direct-zol RNA MicroPrep kits were obtained from Zymo Research (Cat# R2060).

### Animals

All animal studies were approved by the University of Pittsburgh Medical Center Institutional Animal Care and Use Committee, which adhere to the National Institutes of Health Guide for the Care and Use of Laboratory Animals and reported in accordance with the Animal Research: Reporting *In Vivo* Experiments (ARRIVE) guidelines 2.0 (Percie du Sert et al., 2020). The C57Bl/6J strain mice (male and female, 2–3 months old) used in the study were purchased from Jackson laboratories (Bar Harbor, ME). Mice were housed in a temperature-controlled room on a 12-h light/12-h dark cycle with standard mouse diet and water *ad libitum*.

### Transient ischemic stroke model

Ischemic stroke was induced by transient middle cerebral artery occlusion (tMCAO) and reperfusion as previously described (Begum et al., 2015). Briefly, mice were anesthetized in an induction chamber filled with gas mixture (3% isoflurane vaporized in 60% N_2_O and 40% O_2_) for 5 min and then decreased to 1.5% isoflurane for maintenance during the surgery period. Under an operating microscope, the hair was removed and a midline pre-tracheal incision was made to expose the anterior cervical area. The left common carotid artery and the external carotid artery (ECA) were isolated and ligated. After isolation of the internal carotid artery (ICA) while avoid excessive stretching of adjacent vagus nerve, a monofilament suture coated with rubber silicon (size 6–0, diameter with coating 0.21 ± 0.02 mm; coating length 5–6 mm) was inserted into the ECA then advanced into the ICA 8 mm away from the bifurcation of the carotid artery. The ECA stump was ligated with a silk suture to prevent bleeding. After 50 or 60 min blockage of MCA blood flow, the suture was withdrawn slowly and gently for blood flow reperfusion. The sham surgery mice received the same operation except the insertion of the monofilament suture. A small heating pad was used to maintain mouse body temperature between 36.5 ± 0.5°C during the entire procedure.

### Cerebral blood flow (CBF) measurement

Relative CBF changes (rCBF) were measured by a two-dimensional laser speckle contrast analysis system (PeriCam PSI High Resolution with PIMSoft, Perimed, Sweden). Mice were anesthetized in mixed gas containing 70% N_2_O, 30% O_2_ and 1.5% isoflurane. A midline incision was made in the scalp and the exposed skull surface was cleaned with sterile normal saline. A camera was placed 10 cm above the skull. Raw images of the whole brain were taken under the same camera settings as described before (Zhang et al., 2020). Regional CBF values (arbitrary perfusion units) were measured for the same size of region of interest (ROI) area in both parietal lobes at baseline, 15–20 min, and 24 h post-surgery. The percentage change of rCBF was calculated by comparing to the baseline. Body temperature was maintained at 36.5 ± 0.5°C with a heating pad throughout surgery.

### Stroke infarction volume quantification with 2,3,5-Triphenyltetrazolium chloride (TTC) staining

At 24 h of reperfusion, mice were euthanized in CO_2_ chamber and then decapitated. Total 4 coronal brain tissue slices (2 mm) were stained for 20 min at 37 °C with 1% TTC (Sigma, St. Louis, MO, USA) in PBS solution. Infarction volume was calculated with correction for edema using ImageJ software as described before (Swanson et al., 1990; Bhuiyan et al., 2017).

### Immunofluorescent staining and image analysis

Mice were euthanized in CO_2_ chamber and transcardially perfused with 50 mL ice-cold normal saline, followed by 40 mL ice-cold 4% paraformaldehyde (PFA) in 0.1 M PBS as described before (Song et al., 2018). Brains were dissected and kept in 4% PFA for fixation. A total of 24 h later brains were transferred into 30% sucrose solution for cryoprotection. Coronal brain sections (25-μm thick, at + 0.62 to −0.82 mm posterior from Bregma) were selected for staining. After 3 times washing in TBS buffer, brain sections were incubated with blocking solution (10% normal goat serum (NGS) + 0.5% Triton X-100 in TBS) for 1 h at room temperature (RT) followed by incubation with primary antibodies (Additional file 1: Supplementary Table 1) diluted in the TBS + + solution (3% NGS and 0.3% Triton X-100 in TBS) at 4°C overnight. On the following day, brain sections were washed three times in TBS and then incubated with respective secondary antibodies: goat anti-rabbit Alexa 488-conjugated IgG (1:200), goat anti-rabbit Alexa 546-conjugated IgG (1:200), goat anti-mouse Alexa 546-conjugated IgG (1:200) at RT for 1 h. After washing for 3 × 5 min, DAPI were applied for nuclei staining (1:1000 dilution in the TBS + + solution) for 15 min at RT. Sections were mounted with Vectashield mounting medium (Vector Laboratories, Burlingame, CA, USA). For negative controls, brain sections were stained with the secondary antibodies only (Additional file 1: Supplementary Figure 1). Fluorescent images were captured with Nikon A1R or Nikon A1R SIM inverted microscope with a FV1000 laser scanning confocal system using a 40x oil-immersion objective. Identical digital imaging acquisition parameters were used and analyzed by a blinded observer throughout the study. Fluorescence images were quantified with Fiji (NIH) software.

### ChP preparation and flow cytometry analysis

Mice were euthanized with overdose of CO_2_ and intracardially perfused with ice-cold phosphate buffered saline (PBS) (Song et al., 2018). Ipsilateral (IL) and contralateral (CL) lateral ventricle ChP (LVCP) tissues were isolated from two mouse brains and pooled in each experiment. LVCP were incubated with enzyme mix solution (1X HBSS buffer containing 1000 U/ml collagenase I, 400 U/ml collagenase IV, 100 μg/ml DNAase I and 3 mM CaCl_2_) for 45 min at 37°C and manually dissociated into single-cell suspension by pipetting. Cells were incubated for 20 min at 4°C with antibodies against different immune cell surface markers (Additional file 1: Supplementary Table 1). Data were acquired using an LSRII flow cytometer equipped with FACS Diva program and analyzed with FlowJo software.

### Immunoblotting of ChP tissues

Mice were anaesthetized and perfused with ice-cold PBS. IL and CL LVCP tissues were isolated from two mouse brains and pooled in each experiment. ChP tissues were transferred into ice-cold Pierce RIPA buffer containing 1X Halt protease inhibitor cocktail (Thermo Scientific) and 1X PhosSTOP phosphatase inhibitor cocktail (Roche). Tissues were homogenized by pipetting and cellular debris were removed by centrifugation at 10,000 × *g* for 10 min at 4°C and protein concentrations were measured using a BCA protein assay kit. Total protein (∼30 μg/well) in 4 × Laemmli reducing sample buffer (Bio-Rad, Hercules, CA, USA) was heated at 95°C for 5 min before loading onto 4–20% gels. Sodium dodecyl sulfate-polyacrylamide gel electrophoresis (SDS-PAGE) was run using the Mini Protein 3 System (BioRad) and separated proteins were transferred onto 0.2 μm nitrocellulose membranes (BioRad). The membranes were blocked with 1% BSA in PBS containing 0.05% Tween 20 (PBST) at RT for 1 h and were incubated with the appropriate primary and secondary antibodies. Secondary antibody was labeled with either IRDye 680LT (for 680 nm) or 800CW (for 780 nm). Different fluorescent signals from specific proteins were scanned and discriminated using two independent infrared detection channels at 685 and 785 nm excitation wavelengths of Odyssey Infrared Imaging System (Li-Cor, Lincoln, NE, USA), which were quantified by the NIH ImageJ software.

### Real-time reverse transcription polymerase chain reaction (RT-PCR) of ChP tissues

Lateral ventricle choroid plexus from each brain was isolated and ChP pellet was obtained via centrifugation at 300 × *g* (4°C) for 10 min. Total RNA of ChP was extracted using the Direct-zol RNA MicroPrep kit. RNA was quantified under spectrophotometer ND-1000 (NanoDrop) by measuring absorbance. All RNA was converted into cDNA in the Thermo cycler (25°C 5 min, 42°C 30 min, 85°C 5 min, cool down to 4°C) and synthesis of cDNA was performed by using iScript Reverse Transcription Supermix. Quantitative RT-PCR was performed using iTaq Universal SYBR Green Supermix. Sequences of forward and Reverse primers are listed (Additional file 1: Supplementary Table 2). RT-PCR analysis was performed for relative expression changes of target mRNAs over Gapdh reference gene by using 2^^(–ΔΔCt)^ method.

### ELISA measurement of albumin in CSF and serum

Blood sample (0.5 ml) in the test tube was obtained by cardiac puncture from isoflurane anesthetized mice and left at RT for 30 min for clotting. After centrifuge at 1,500 × *g* (4°C) for 15 min, the serum layer was collected. CSF sample was collected from cisterna magna by fixing mouse head by ear bars on a stereotaxic instrument to form a 135° angle between the head and body. A capillary was punctured into the cisterna magna and 5 μL CSF was withdrawn. After centrifugation at 1,500 × *g* (4°C) for 10 min, the CSF supernatant was stored at −80°C. Albumin concentrations in CSF and sera were measured by using RayBio mouse albumin ELISA kit (RayBiotech, Catalog# ELM-Albumin) with the dilution of CSF (1:2000) and serum (1: 1.5 × 10^6^). The assay was performed according to the manufacturer’s instructions. The values of CSF_*Albumin*_/Serum_*Alumin*_ ratio were used for the assessment of blood-CSF brain barrier permeability changes.

### Statistical analyses

**FIGURE 1:**
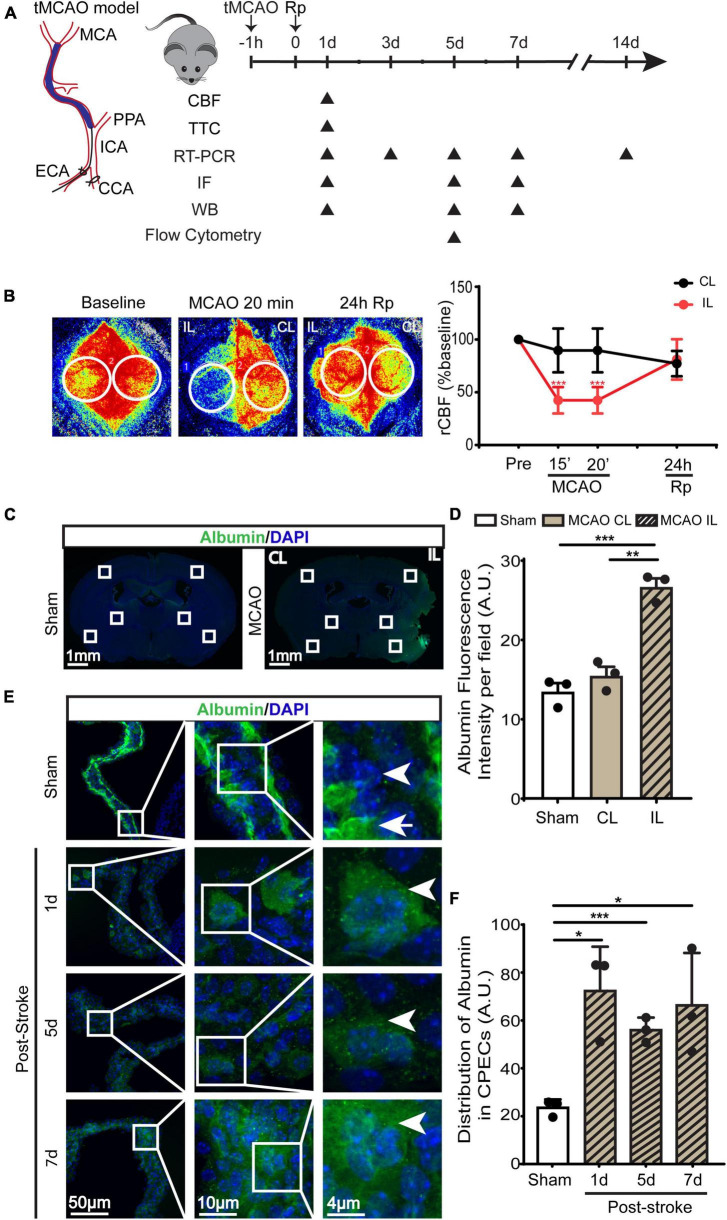
Transient ischemic stroke-induced hypoperfusion and the blood-CSF barrier damage in C57BL/6J mice. **(A)** Experimental protocol. Transient middle cerebral artery occlusion (tMCAO) was used to induce ischemic stroke in C57BL/6J mice. **(B)** Representative images of relative cerebral blood flow (rCBF) change and quantification via Peri-CAM PSI. Region of interest (ROI): circle. CL, contralateral hemisphere; IL, ipsilateral hemisphere. Data were mean ± SEM. *n* = 6. ***p < 0.001 CL vs. IL. **(C)** Representative epifluorescence images of albumin protein immunofluorescence staining at 1-, 5-, or 7- day post-stroke or sham surgery. **(D)** Quantification of peri-infarct albumin intensity. Data are mean ± SEM. **(E,F)** Representative confocal immunofluorescence images of ChP and quantification data of average ROI albumin intensity. Arrowhead: cellular albumin. Arrow: vascular albumin. Data are mean ± SEM, *n* = 3. *p < 0.05; **p < 0.01; ***p < 0.001.

**FIGURE 2:**
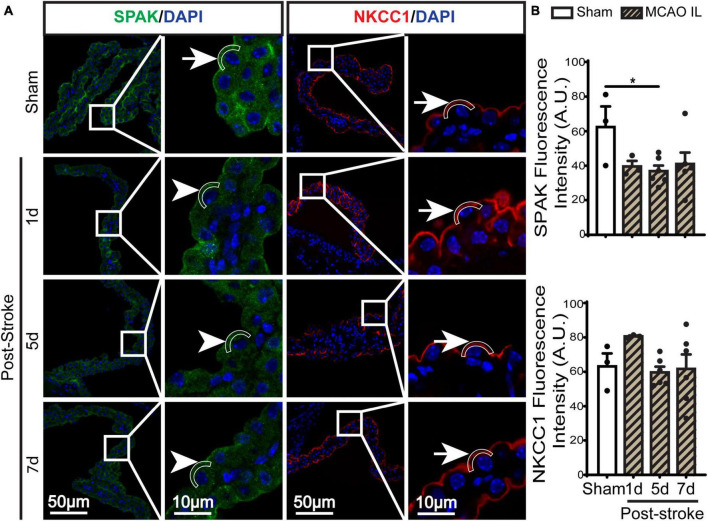
Dynamic changes of SPAK-NKCC1 complex expression in ChP after ischemic stroke. **(A)** Representative confocal immunofluorescence staining images of SPAK and NKCC1 protein in the LVCP (IL: ipsilateral) at 1-, 5-, or 7-day post-stroke or sham surgery. Arrow: high expression. Arrowhead: low expression. **(B)** Quantification for fluorescence intensity of apical SPAK and NKCC1 protein in ROI. Data are mean ± SEM, *n* = 3–6. *p < 0.05.

**FIGURE 3:**
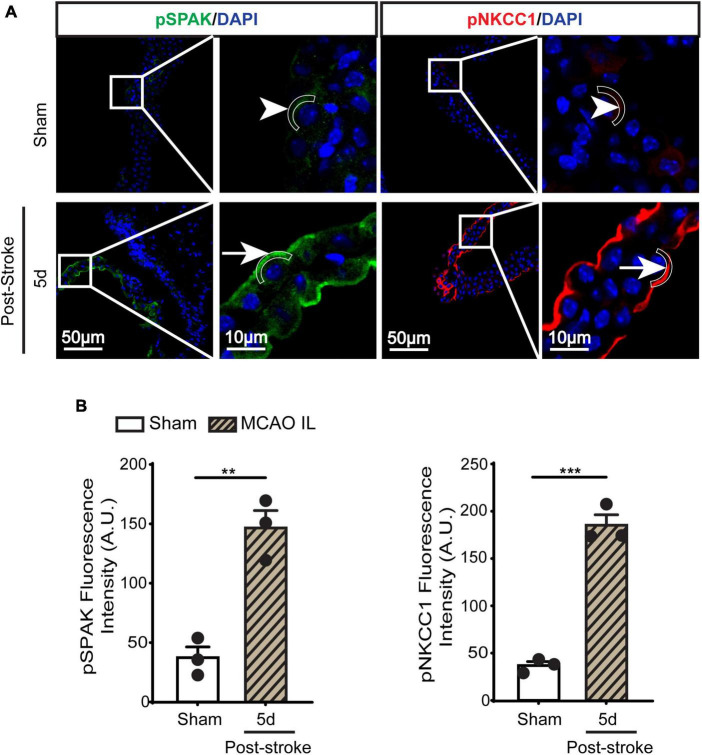
Dynamic changes of SPAK-NKCC1 complex phosphorylation in LVCP after ischemic stroke. **(A)** Representative confocal immunofluorescence staining images of phosphorylated SPAK and NKCC1 proteins in LVCP (IL: ipsilateral) at 5-day post-stroke or sham surgery. Arrow: high expression. Arrowhead: low expression. **(B)** Quantification of pSPAK and pNKCC1 fluorescence intensity. Data are mean ± SEM, *n* = 3. **p < 0.01; ***p < 0.001.

**FIGURE 4:**
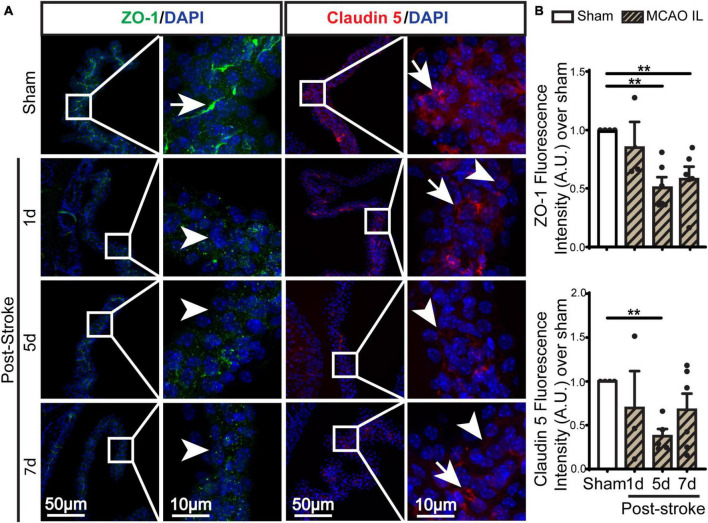
Dynamic changes of TJ proteins in LVCP after ischemic stroke. **(A)** Representative confocal immunofluorescence staining images of ZO-1 and Claudin 5 proteins at 1-, 5-, 7-day post-stroke (IL: ipsilateral) or sham surgery. Arrow: high expression. Arrowhead: low expression. **(B)** Quantification of ZO-1 and Claudin 5 fluorescence intensity. Data are mean ± SEM, *n* = 3–6. **p < 0.01.

**FIGURE 5:**
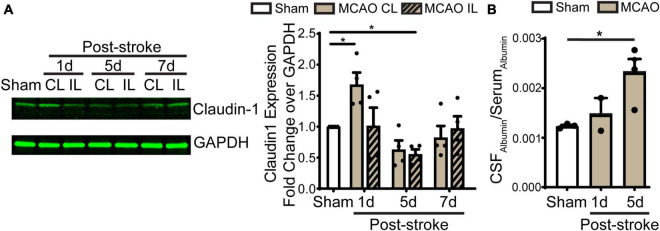
Transient ischemic stroke induces dynamic changes of claudin-1 in LVCP and the blood-CSF barrier leakage. **(A)** Representative immunoblot image of claudin-1 protein band and quantification of LVCP samples at 1-, 5-, and 7-day post-stroke or Sham. Data are mean ± SEM, *n* = 4. *p < 0.05. **(B)** Quantification of albumin in CSF and serum by ELISA. Data are mean ± SEM, *n* = 3 in sham, *n* = 2 in 1 days post-stroke, *n* = 4 in 5 days post-stroke. *p < 0.05.

**FIGURE 6:**
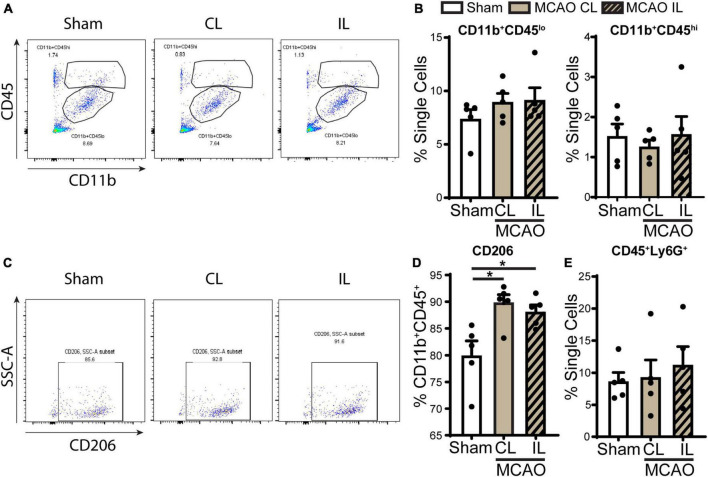
Flow cytometry analysis of immune cell infiltration in the ChP after ischemic stroke. **(A)** Representative flow cytometric plots of CD11b^+^CD45^+^ myeloid cells in ChP at day 5 post-stroke or sham. CL, contralateral LVCP; IL, ipsilateral LVCP. **(B)** Quantification for percentage of CD11b^+^CD45^lo^ and CD11b^+^CD45^hi^ myeloid cells in singlets. **(C,D)** Representative flow cytometric plots and the percentage of CD206^+^ cells gated within CD11b^+^CD45^+^ cells. **(E)** Percentage of CD45^+^Ly6G^+^ neutrophils in the ChP gated with single cells. Data are mean ± SEM (*n* = 4–5), *p < 0.05.

**FIGURE 7:**
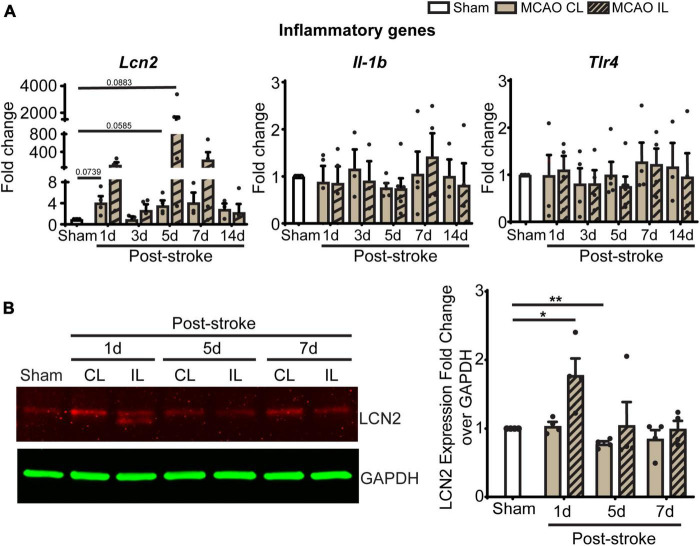
Transient ischemic stroke induces sustained inflammatory transcription changes in ChP. **(A)** Relative expression changes of proinflammatory gene mRNAs was performed with RT-PCR analysis over *Gapdh* reference gene. Data are mean ± SEM. Sham: *n* = 10, Stroke: *n* = 4∼6. CL, contralateral LVCP; IL, ipsilateral LVCP. **(B)** Representative LCN2 protein band and summary analysis from LVCP samples at 1-, 5-, and 7-day post-stroke or Sham surgery were shown. Data are mean ± SEM. *n* = 4. *p < 0.05, **p < 0.01.

A total of 155 C57Bl/6J (male, and female) mice were used in this study (Additional file 1: Supplementary Table 3). A total of 6 mice were excluded due to the failed surgeries. A total of 17 mice died after tMCAO stroke, resulting in a mortality rate around 14%. All data presented in this study was original, no data sharing or pooling from any previous published or non-published studies included. An investigator who was blind to experimental group assignments performed data assessments. Unpaired *t*-test was performed to compare each experimental group (1, 5, and 7 days) with sham control, respectively, for data in Figures 1–3, 6. One sample *t*-test comparison with a hypothetical mean was used for data analysis in Figures 4, 5, 7 and Supplementary Figure 2. *N-*values represent the number of independent experiments. Data were expressed as mean and standard error of mean (SEM). We considered statistically significant when a *p*-value is lower than 0.05. Data were plotted using GraphPad Prism 9 (GraphPad Software, Inc., CA, USA).

## Results

### Stroke causes cerebral hypoperfusion, infarction, and loss of the blood-CSF barrier integrity

Transient ischemic stroke in C57BL/6J mice was induced by 60-min transient middle cerebral artery occlusion (tMCAO) (Figure 1A). PeriCam PSI detected ∼10% decrease in rCBF in the CL cortex and ∼50% decrease of rCBF in the IL cortex after MCAO (Figure 1B). By 24 h reperfusion (Rp), rCBF of the CL and the IL cortices recovered to the pre-stroke baseline levels (Figure 1B). This resulted in an average of ∼80 mm^3^ total infarct stroke volume (TTC staining analysis), with ∼40 mm^3^ infarct in the cerebral cortex and ∼30 mm^3^ in the striatum. As shown in Figures 1C, D (4 × low mag images), we first assessed the leakage of blood-brain barrier by immunostaining of albumin for its infiltration into brain parenchyma in peri-infarct area in the ipsilateral (IL) and contralateral (CL) hemispheres. It shows that sham or the CL hemisphere of stroke brains displayed minimum albumin infiltration. In contrast, a significant increase of albumin infiltration was detected in the IL peri-infarct area of stroke brains, reflecting a loss of blood-brain barrier integrity. In the case of ChP, in Sham LVCP, majority of albumin immunostaining signals were confined in the stroma blood vessels (Figure 1E, arrow) with less albumin immunostaining signals detected in the cytosol of CPEC (arrowhead). This polarized albumin distribution in CPECs and blood vessels has been well documented (Liddelow et al., 2014). However, the stroke ChP showed loss of overall retention of albumin in the blood vessel but increased albumin immunosignals in the cytosol of CPEC. This altered albumin permeability at the blood-CSF barrier was further analyzed by calculating the intracellular albumin intensity in CPECs, showing a 2–3-fold increase in the IL LVCP at 1–7 days post-stroke (Figure 1F, *P* < 0.05). These data suggest that ischemic stroke caused sustained increase of the blood-CSF barrier damage.

### Stroke-induced changes of ChP SPAK-NKCC1 protein complex expression and phosphorylation

SPAK (Ste20-related proline-alanine-rich kinase)/OSR1 (oxidative stress-responsive kinase-1) are essential for regulating NKCC1 protein and are associated with NKCC1 protein activation in the lipopolysaccharides (LPS)-induced hydrocephalus (Huang et al., 2019; Robert et al., 2023). We examined changes of SPAK-NKCC1 protein complex in CPECs by immunostaining. As shown in Figures 2A, B, Sham CPEC displayed abundant expression of SPAK at the apical membrane (arrow). At 1–7 days post-stroke, there was a continuous reduction of SPAK protein expression (arrowhead) in IL LVCP tissues, which showed significance at 5 days post-stroke (*p* < 0.05). In the case of NKCC1 protein, there was an elevation in CPECs at 1 day post-stroke and which returned to the normal level at 5–7 days after stroke, and this did not reach statistical significance. In contrast, significant elevation of pSPAK and pNKCC1 in the apical membrane of CPECs was detected at 5 days post-stroke in the IL LVCPs (Figure 3). Interestingly, similar changes of SPAK-NKCC1 complex were detected in the CL LVCP of stroke brains (Supplementary Figures 3, 4). Taken together, these findings demonstrate that stroke triggered dynamic changes of SPAK-NKCC1 protein complex expression and phosphorylatory stimulation in the subacute stage.

### Loss of CPEC TJ proteins and the blood-CSF permeability after stroke

Previous study shows ischemic stroke causes reduction of occludin, claudin-1, and ZO-1 of the blood-brain barrier at 14 days post-stroke (Chen et al., 2021). We then assessed whether stroke induces changes of the blood-CSF barrier TJ integrity by probing for ZO-1 and claudin 5 protein expression by immunostaining. As shown in Figures 4A, B, sham ChP displayed abundant ZO-1 and claudin 5 proteins at the CPEC cell-cell interface (arrow). In contrast, the reduction in both ZO-1 and claudin 5 were detected at the 1–7 days post-stroke, both was significant at 5 days after stroke (Figure 4B. *p* < 0.01). We further examined changes of claudin-1 protein in LVCPs in sham or stroke mice at 1, 5, and 7 days post-tMCAO by Western Blotting. As shown in the Figure 5A, ischemic stroke triggered an initial elevation of claudin-1 protein at 1 day post-tMCAO, followed with a significant loss of claudin-1 protein expression by 5 days post-tMCAO (*p* < 0.05). Claudin-1 protein expression was then returned to the control level by 7 days post-stroke. This finding demonstrated different sensitivity to stroke-induced damage and dynamic recovery of the TJ proteins (claudin-1 and claudin-5, and ZO-1) at the blood-CSF barrier epithelium. Further, to assess leakage of albumin into the CSF, we performed ELISA to measure the amount of albumin in CSF and serum of the sham control and tMCAO mice at 1 and 5 days post-stroke. The ratio of CSF_*Albumin*_/Serum_*Albumin*_ increased by twofold in the 5 days post-stroke mice when compared to sham (Figure 5B, *P* < 0.05). This is associated with the significant loss of TJ protein claudin-1 at 5 days post-stroke as shown in Figure 5A. These data collectively demonstrated ischemic stroke-induced damage of the blood-CSF barrier and increased permeability.

### Flow cytometry analysis of immune cell infiltration in ChP

We next assessed the infiltration of immune cell in the ChP after stroke by flow cytometry. Probing of ChP isolated from 5 days post-stroke brains revealed that there were no significant changes of CD11b^+^CD45*^lo^* (microglia) or CD11b^+^CD45*^hi^* (peripheral macrophages) myeloid cell counts compared to sham (Figures 6A, B). Interestingly, gating within the CD11b^+^CD45^+^ cells, we found that the CD206^+^ population cells (alternatively activated beneficial myeloid cells) were significantly increased in the 5 days post-stroke ChP compared to the sham control (Figures 6C, D, *p* < 0.05). There is no statistically significant difference in the percentage of CD45 + /Ly6G + neutrophils between 5 days post-stroke ChP and sham ChP (*p* = 0.425). Taken together, these data illustrate that at the 5 days post stroke, ischemic stroke-mediated proinflammatory damage of ChP is subsided and that there is simultaneous restorative activation of the CD11b^+^CD45^+^ cells/CD206^+^ population myeloid cells for tissue repair, which is consistent with previous reports (Rayasam et al., 2020).

### Transient ischemic stroke induces sustained proinflammatory ChP gene transcription changes

In light of these changes of immune cells in ChP, we further screened gene transcription changes of key ChP genes including ChP proinflammatory genes and the WNK-SPAK-NKCC1 complex at 1, 3, 5, 7, and 14 days post-stroke. Compared to Sham, post-stroke ChP did not display statistically significant changes in either inflammatory genes or the *Wnk-Spak-Nkcc*1 pathway genes (Figure 7A and Supplementary Figure 2). The *Lcn*2 gene [encoding for lipocalin-2 (LCN2)] showed the trend of upregulation at 1 and 5 days in post-stroke ChP where the *p*-value was close to 0.05 (*p* = 0.0585).

Lipocalin-2, also known as neutrophil gelatinase associated lipocalin (NGAL), is a 25 kDa protein that involved in immune infiltration, brain edema, neuronal death, and neurological deficits (Zhang et al., 2022). Previous studies suggest that ChP is one of the main sources of LCN2 after systemic lipopolysaccharide administration (Ip et al., 2011). We performed immunoblotting to assess ChP LCN2 protein expression changes after 1, 5, and 7 days of tMCAO. The CL LVCP showed no differences in LCN2 protein levels compared to sham mice at day 1–7 post-stroke. However, the IL LVCP displayed significant increase of LCN2 at day 1, which gradually decreased during days 5 and 7 post-stroke (Figure 7B, *P* < 0.05). These results parallel the qRT-PCR results. Taken together, the changes in inflammation-related LCN2 protein may be a pathway for the increased permeability of the blood-CSF barrier and myeloid cell infiltration.

## Discussion

### Ischemic stroke-induced persistent damage of the blood-CSF barrier at 1–7 days post-stroke

Choroid plexus in the ventricles of the brain has an imperative role in regulating the CNS (Dziegielewska et al., 2001). The ChP produces the CSF that acts as a clearance mechanism for the brain, gives rise to the blood-CSF barrier that protects the brain from pathogens, and acts as an immune recruitment site during times of need (Redzic and Segal, 2004; Solár et al., 2020). With increasing impact of neuroinflammation on brain pathologies (Roth et al., 2021), it is important to understand how the ChP changes in pathological conditions, including ischemic strokes. Researchers have explored several ischemic models to study ChP structural and functional changes after stroke. One study shows that permanent MCAO in rat without a tandem CCA occlusion causes 38% reduction in LVCP blood flow but results in LVCP edema at 24 h post-stroke (Ennis and Keep, 2006). Transient MCAO in rats also revealed sustained CPEC death at 1–28 days post-stroke (Li et al., 2002). Moreover, DNA fragmentation were observed in rat ChP at 18–24 h following 15 min of forebrain ischemia (Ferrand-Drake and Wieloch, 1999; Ferrand-Drake, 2001). Most of these studies were focused on changes of ChP during the acute 1–2 days post-stroke period. In our study, we used mouse tMCAO stroke model to further investigate post-stroke changes in the ChP cytoarchitecture structures at 1–7 days post-stroke. We found that there is a sustained breakdown of the blood-CSF barrier in the mouse ChP at 1–7 days subacute post-stroke stage, which was reflected by CPEC uptake and/or leakage of blood plasma albumin into CSF during the post-stroke subacute period (days 1–7). Evidence for the disintegration of the barrier was also supported by the corresponding loss of TJ proteins, ZO-1, claudin 5, and claudin-1, found between the CPECs (Figures 4, 5). An important component of the ChP with relevance to this is SPAK-NKCC1 protein complex, the ion transport regulatory apparatus that is responsible for the ChP’s CSF production and clearance (Steffensen et al., 2018; Gregoriades et al., 2019; Blazer-Yost, 2023). NKCC1 is regulated by SPAK kinase, which phosphorylates it for activation after generating a NKCC1-SPAK complex (Blazer-Yost, 2023). Our previous study has shown that SPAK and NKCC1 protein expression pathologically increased at 24 h after tMCAO (Wang et al., 2022). Our new results displayed a dynamic change of SPAK-NKCC1 protein complex during the subacute period after tMCAO, with a decrease of SPAK protein over days 1–5 after a stroke, but a sustained increase in pSPAK and pNKCC1 protein in the apical membrane of CPECs. An increased phosphorylation state of both protein complexes indicates higher activation of pSPAK and pNKCC1. In disease conditions, activated SPAK has an important role in converting stress and cytokine inflammatory signaling, including TLR4 and NF-κB dependent activation, into NKCC1-mediated hyper- or pathological secretion of CSF (Robert et al., 2023). Other research in the field has also linked changes to these protein complexes to ChP CSF production dysregulation (Podvin et al., 2011; Jang et al., 2022). Further studies will be needed to investigate impact of increased phosphorylation states of SPAK and NKCC1 on ChP CSF regulation in ischemic stroke brains.

In this study, despite of ipsilateral stroke was induced in the MCAO model, changes of ChP were detected in both CL and IL LVCP tissues. Such a phenomenon has been observed in previous reports. For example, in an intracerebral hemorrhage model, whole blood injection into one hemisphere striatum induced gene expression changes (MyD88-TLR4 signaling) and immune cells infiltration in both IL and CL ChPs (Akeret et al., 2022). Elevation of pN-κB was detected in both IL and CL ChPs of ischemic stroke brains induced by tMCAO in our recent study (Wang et al., 2022). Thus, activation of inflammatory responses via cytokines in the CSF may result in bilateral LVCP damage in our focal ischemic stroke model.

### Myeloid cell infiltration into post-stroke ChP

In addition to the increased susceptibility to microbes from the body, there can be excess inflammation in the brain with a greater volume of infiltrating immune cells and their secreted signaling molecules (Ge et al., 2017; Llovera et al., 2017). During normal brain functioning, the ChP serves as a site of immune surveillance and storage of immune molecules (Municio et al., 2023). There is evidence that ChP volume is larger in people who have experienced strokes, compared to healthy controls (Egorova et al., 2019). The ChP stroma has reserves of various immune molecules including macrophages, basophils, neutrophils, B cells, and T cells (Dominguez-Belloso et al., 2022). After an ischemic stroke, the ChP recruits a greater volume of immune cells and molecules to the site of the injury, particularly T lymphocytes (Llovera et al., 2017). In this study, we assessed immune cell infiltration into ChP by flow cytometry. There were not significant increases in CD45^+^/Ly6G^+^ immune cell infiltration into the ChP by 5-day post-stroke compared to sham. However, a significantly high count of CD206-expressing CD45^+^/CD11b^+^ myeloid cells was detected in the 5-day post-stroke ChP tissues, suggesting anti-inflammatory restorative immune response process. This is a different finding from the pro-inflammatory myeloid cell recruitment peaking at 3 days post-stroke reported in a previous study (Muhammad et al., 2021). Future studies are needed to probe the temporal profiles of infiltrated immune cells in the ChP in acute and subacute post-stroke time points, which will enable us to better understand the blood-CSF barrier damage and repair after ischemic stroke.

### Ischemic stroke-induced proinflammatory LCN2 protein elevation at ChP

Lastly, in assessing cellular factors which possibly cause stroke-mediated ChP damage, we identified a gene and protein upregulation in LCN2, a critical component of the ensuing inflammatory response. LCN2 is an acute phase molecule released by various tissues in the body that has been linked to injury, infection, and overall inflammation (Zhang et al., 2022). In addition, there is evidence that LCN2 may be linked to worse outcomes post stroke. tMCAO mice with LCN deficit (LCN2^–/–^) had reduced infarct volume and less amounts of other proinflammatory molecules while mice with LCN overexpression had larger infarct volumes and worsened behavioral outcomes (Zhang et al., 2022). Knowing that LCN2 signaling damages both neurons and the blood-brain barrier, LCN2 expression in brain tissue and in circulation after harmful conditions may be the direct cause of the brain injury that follows a stroke (Zhang et al., 2022). In our study, higher LCN2 mRNA level than LCN2 protein was detected in LVCPs in post-stroke brains. We speculate that this is likely due to the secretion of LCN2 protein from ChP epithelial cells into the CSF, which has been reported in systemic lipopolysaccharide administration-induced inflammation (Marques et al., 2008; Ip et al., 2011). In addition, it is possible that elevated LCN2 mRNA expression may reflect a feedback mechanism for compensating low LCN2 protein level at the ChP. On whether elevated LCN2 at the blood-CSF barrier plays a role in triggering the breakdown of the ChP blood-CSF barrier, SPAK-NKCC1 dysregulation, and immune cell infiltration to ChP remains to be investigated in future studies.

In this study, we used both male and female in most experiments (Supplementary Table 3) and did not observe differences in mortality rates or changes in ChP damages between male and female mice. However, limitation of our study is that *n*-values are too small to evaluate sex as a factor in this study.

**FIGURE 8:**
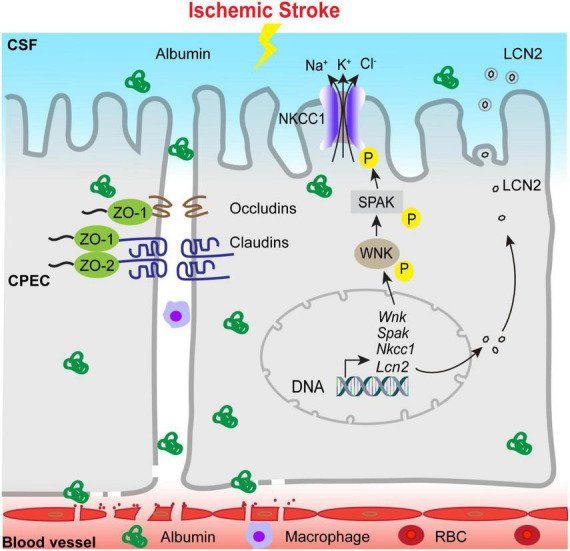
Schematic illustration of ischemic stroke-induced changes of the ChP blood-CSF barrier integrity. Ischemic stroke triggers the damage of blood-CSF barrier and results in permeability increase which lasts at least 7 days post-stroke, which reflected by uptake of the albumin by CPECs, the breakdown of TJs and infiltration of circulating immune cells, etc. Ischemic stroke also causes upregulation of proinflammatory *Lcn2* gene and LCN2 protein expression especially in the early acute post-stroke stage, accompanied with sustained reduction of choroidal SPAK-NKCC1 protein expression but activation of SPAK as well as NKCC1 phosphorylation. Ischemic stroke also induced infiltration of CD45^+^/CD11b^+^ myeloid cells into the ChP. Taken together, stroke-mediated damages of the ChP may lead to inflammation and dysregulation of CSF.

In summary, we report that ischemic stroke in C57BL/6J mice triggers time-dependent changes in the blood-CSF barrier permeability, tight-junction damage and dynamic changes of SPAK-NKCC1 complex pathway at 1–7 days post-stroke, reflected by an elevation of albumin permeability, transient increase of SPAK-NKCC1 protein phosphorylation, elevation of proinflammatory *Lcn2* mRNA and protein expression, and immune cell infiltration into the ChP (Figure 8). These findings indicate that ischemic stroke causes sustained damage of the ChP blood-CSF barrier in the subacute stroke brains. Learning the underlying mechanisms of post-stroke ChP damage and impact on CSF regulation will facilitate developing targeted treatments for brain inflammation in the future.

## Data availability statement

The original contributions presented in the study are included in the article/Supplementary material, further inquiries can be directed to the corresponding authors.

## Ethics statement

The animal study was approved by the University of Pittsburgh Medical Center Institutional Animal Care and Use Committee (IACUC), which adhere to the National Institutes of Health Guide for the Care and Use of Laboratory Animals. The study was conducted in accordance with the local legislation and institutional requirements.

## Author contributions

YC: Data curation, Formal analysis, Methodology, Writing – original draft. LL: Methodology, Writing – review and editing. MB: Methodology, Supervision, Writing – review and editing. KH: Methodology, Supervision, Writing – review and editing. RJ: Methodology, Writing – original draft, Writing – review and editing. SS: Formal analysis, Writing – review and editing. VF: Writing – review and editing. GB: Formal analysis, Methodology, Writing – review and editing. YY: Supervision, Writing – review and editing. DS: Funding acquisition, Resources, Supervision, Writing – review and editing.
