# Supplementary material for: Transient ischemic stroke triggers sustained damage of the choroid plexus blood-CSF barrier
Source: Front Cell Neurosci. 2023 Dec 1;17:1279385. doi: 10.3389/fncel.2023.1279385 (PMC10725199; doi:10.3389/fncel.2023.1279385)
Supplement: Supplementary file 1 [file Data_Sheet_1.docx]

Supplementary Material

Transient ischemic stroke triggers sustained damage of the choroid plexus blood-CSF barrier

Yang Chen, Lin Lin, Mohammad Iqbal H. Bhuiyan, Kai He, Roshani Jha, Shanshan Song, Victoria M Fiesler, Gulnaz Begum, Yan Yin*****, Dandan Sun*****

*** Correspondence:** Dandan Sun: [sund@upmc.edu](mailto:sund@upmc.edu)

Yan Yin: yanyin1208@126.com

# Supplementary Figures

## Supplementary Tables


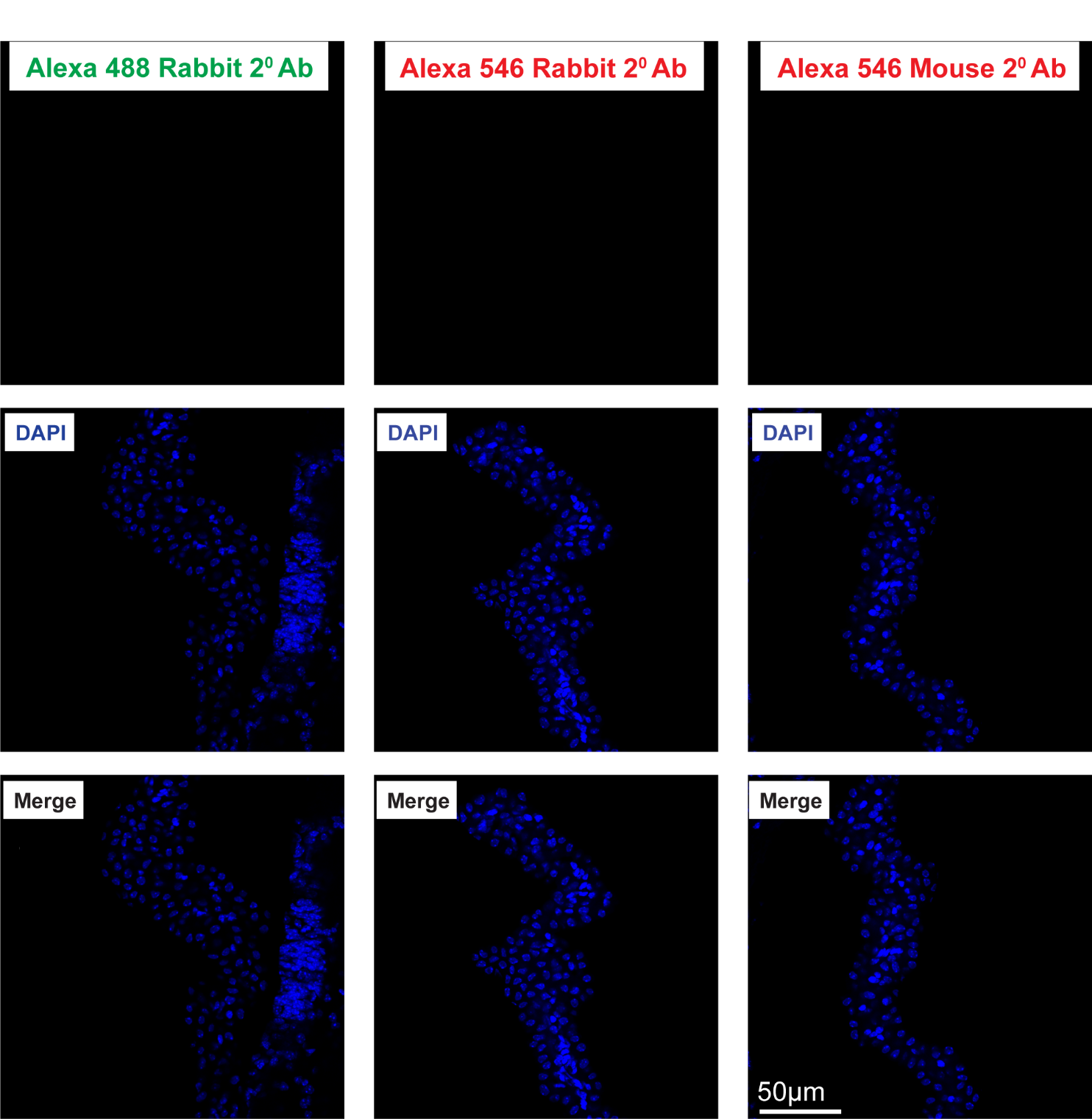


## Figure S1. Immunostaining negative control data

Representative confocal images of mouse LVCP stained with the following secondary antibodies and under identical confocal microscopy settings: Goat anti-rabbit Alexa 488, Goat anti-rabbit Alexa 546 and Goat anti-mouse Alexa 546 at 1:200.

**
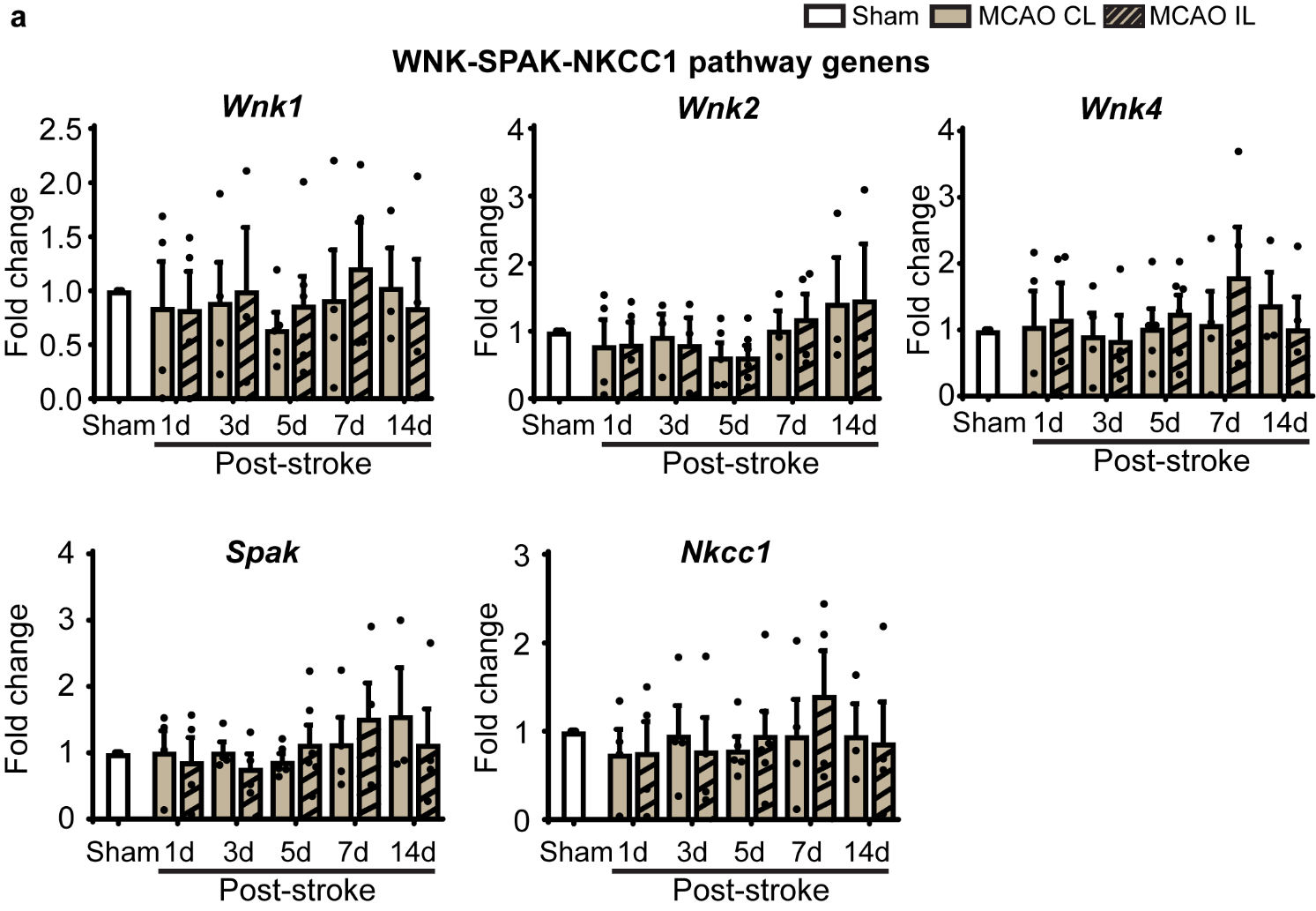
**

**Figure S2.** **Transcription changes of WNK-SPAK-NKCC1 pathway in ChP after ischemic stroke**

**a.** Relative expression changes of WNK-SPAK-NKCC1 pathway gene mRNAs were assessed with RT-PCR analysis over *Gapdh* reference gene. Data are mean ± SEM. Sham: n =10; Stroke: n = 4~6.


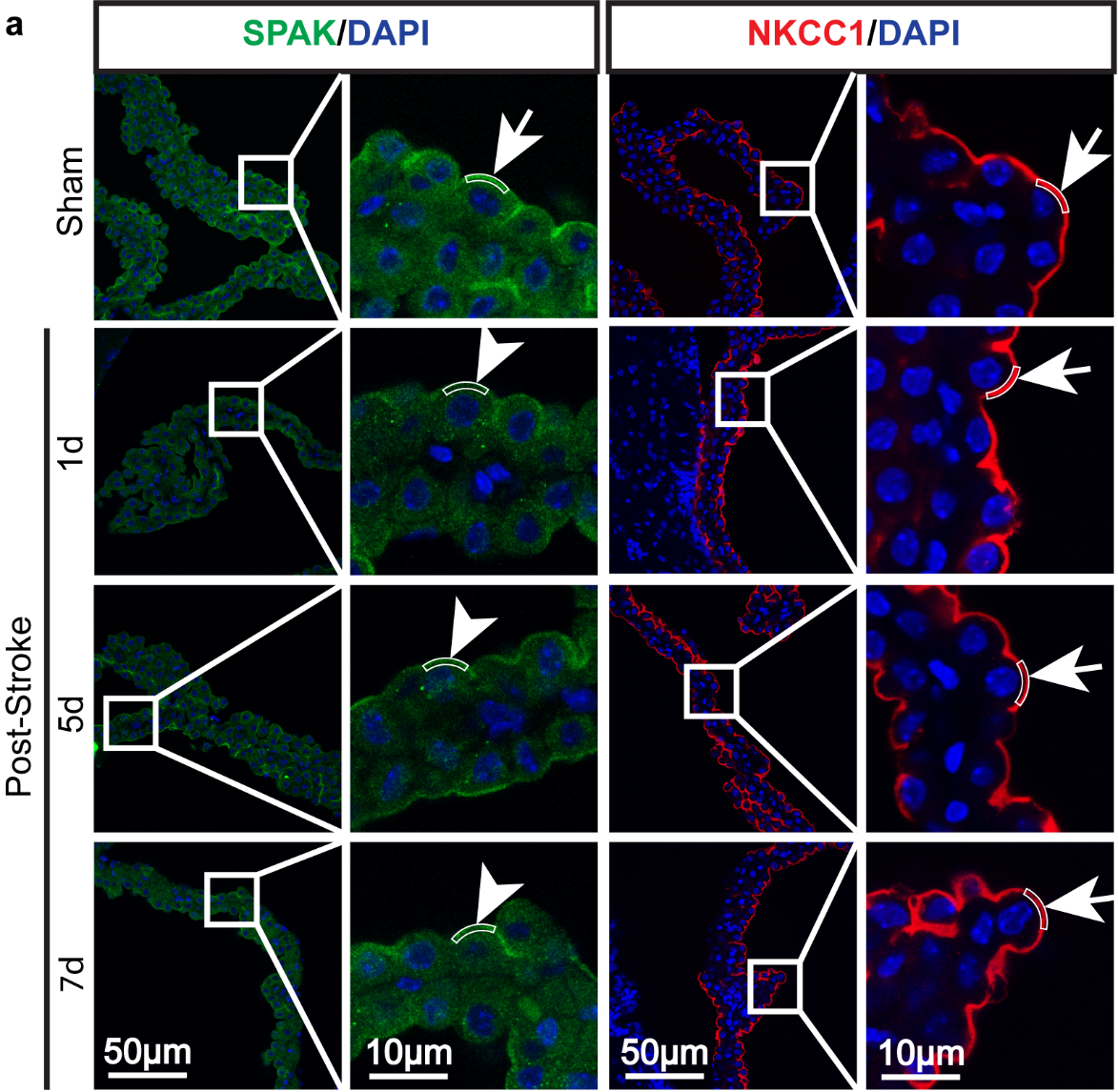


**Figure S3. Dynamic changes of SPAK-NKCC1 complex expression in ChP after ischemic stroke**

**a.** Representative confocal immunofluorescence staining images of SPAK and NKCC1 protein in the LVCP (CL: contralateral) at 1-, 5-, or 7-day post-stroke or sham surgery. Arrow: high expression. Arrowhead: low expression.


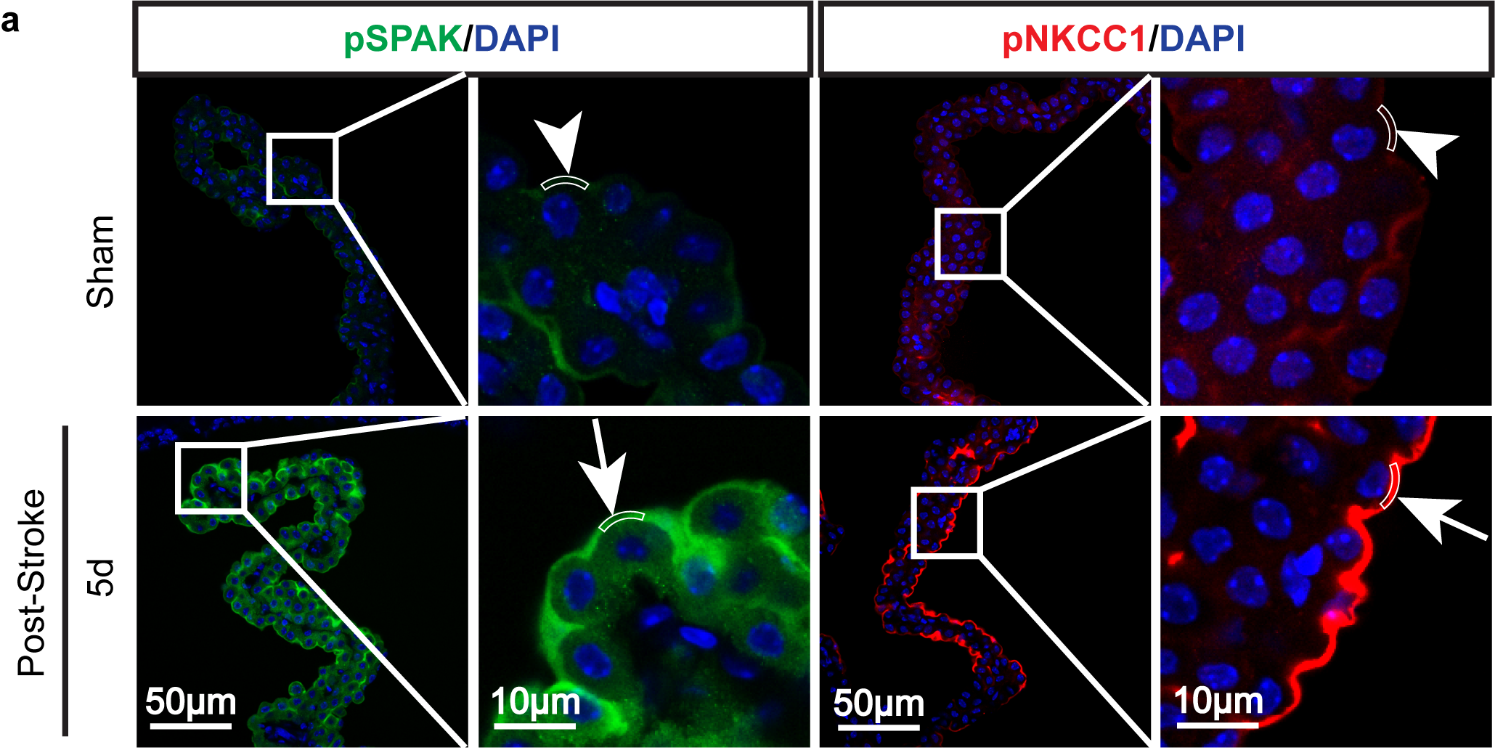


**Figure S4. Dynamic changes of SPAK-NKCC1 complex phosphorylation in LVCP after ischemic stroke.**

**a.** Representative confocal immunofluorescence staining images of phosphorylated SPAK and NKCC1 proteins in LVCP (CL: contralateral) at 5-day post-stroke or sham surgery. Arrow: high expression. Arrowhead: low expression.

**Supplemental Table 1. List of antibodies used for immunofluorescence staining (IF), Western blotting (WB) and flow** **cytometry.**

| **Antibody** | **Host** | **Dilution** | **Company** | **Catalog No.** | **Application** |
| --- | --- | --- | --- | --- | --- |
| SPAK/OSR1 | Rabbit | 1:200 | Cell Signaling | 2281S | IF |
| pSPAK/pOSR1 | Rabbit | 1:200 | EMD Millipore | 07-2273 |  |
| NKCC1(D208R) | Rabbit | 1:200 | Cell signaling | 85403S |  |
| pNKCC1  Albumin  ZO-1  Claudin 5  Alexa anti rabbit 488  Alexa anti rabbit 546  Alexa anti mouse 546 | Rabbit  Rabbit  Rabbit  Mouse  Goat  Goat  Goat | 1:200  1:200  1:100  1:200  1:200  1:200  1:200 | EMD Millipore  abcam  Invitrogen  Invitrogen  Invitrogen  Invitrogen  Invitrogen | ABS1004  Ab19196  40-2200  35-2500  A11008  A11035  A11003 |  |
| LCN 2  Claudin 1  GAPDH | Mouse  Rabbit  Rabbit | 1:1000  1:1000  1:1000 | Santa Cruz Biotechnology  Invitrogen  Cell signaling | sc-515876  51-9000  5174S | WB |
| BUV395-CD11b | Rat | 1:100 | BD Biosciences | 563553 | Flow Cytometry |
| APC-CD45  PE-Cy7-CD206 | Rat  Rat | 1:100  1:100 | BioLegend  Invitrogen | 103111  25-2061-824 |  |
| PerCP/Cy5.5-Ly6G | Rat | 1:100 | BioLegend | 127615 |  |

**Supplemental Table 2. List of primers for RT-PCR**

|  | | **Forward (5’-3’)** | | **Reverse (5’-3’)** |
| --- | --- | --- | --- | --- |
| **G*apdh***  **H*prt***  **I*l1b***  **T*lr4***  **L*cn2***  **W*nk1***  **W*nk2***  **W*nk4***  **S*pak***  **N*kcc1*** | AACTTTGGCATTGTGGAAGG  GCCTAAGATGAGCGCAAGTTG  GACTGCATAGGCTTGCTTCC  TTTATTCAGAGCCGTTGGTG  TGGCCCTGAGTGTCATGTG  CTTTCAGATGGGGCGGTTTT  TTGACAGGGTGCCATACCAT  AGTGCTGCCTTATCAAGGGT  AAGCGCCGGAAAGTTACAAG  GGACTGCATTTCCTAACGCT | | ACACATTGGGGGTAGGAACA  TACTAGGCAGATGGCCACAGG  TGCTTCTGTGCTCTCCATCA  CAGAGGATTGTCCTCCCATT  CTCTTGTAGCTCATAGATGGTGC  GGGATTTGTAGTTTCGCGGT  GATTTCTGAGCCCATGCCAG  CAAGAGCTGAGTGAGTGGGA  GGAGACACCTACCTACACGG  AGAGTAATTCCAGCCGCGAT | |

**Supplemental Table 3. Number of mice used**

| **Experiments** | **Number of Sham mice** | **Number of Stroke mice (MCAO time)** | **Mortality rate** | **Sex**  **(Male, Female)** |
| --- | --- | --- | --- | --- |
| **CBF**  (Fig. 1b) | 0 | 6 (60 min) | 0 | 3 M  3 F |
| **Immunostaining**  (Fig. 1-4, S1, 3-4) | 4 | 19 (50 min & 60 min) | 15.7%  (2 M, 1 F) | 13 M  12 F |
| **RT-PCR**  (Fig. 7a, S2) | 10 | 36 (60 min) | 25%  (6 M, 3 F) | 26 M  22 F |
| **Immunoblotting**  (Fig. 5a, 7b) | 4 | 28 (50 min) | 7%  (2 M) | 32 M |
| **Flow cytometry**  (Fig. 6) | 6 | 14 (50 min) | 14% (2 M) | 16 M  4 F |
| **CSF ELISA**  (Fig. 5b) | 5 | 13 (50 min) | 7.7% (1 M) | 18 M |
